# Making and Maintaining Lifestyle Changes after Participating in Group Based Type 2 Diabetes Self-Management Educations: A Qualitative Study

**DOI:** 10.1371/journal.pone.0064009

**Published:** 2013-05-09

**Authors:** Marit B. Rise, Anneli Pellerud, Lisbeth Ø. Rygg, Aslak Steinsbekk

**Affiliations:** 1 Department of Public Health and General Practice, Norwegian University of Science and Technology, Trondheim, Norway; 2 Helse Nord-Trøndelag Hospital Trust, Levanger, Norway; Edinburgh University, United Kingdom

## Abstract

**Background:**

Disease management is crucial in type 2 diabetes. Diabetes self-management education aims to provide the knowledge necessary to make and maintain lifestyle changes. However, few studies have investigated the processes after such courses. The aim of this study was to investigate how participants make and maintain lifestyle changes after participating in group-based type 2 diabetes self-management education.

**Methods:**

Data was collected through qualitative semi-structured interviews with 23 patients who attended educational group programs in Central Norway. The participants were asked how they had used the advice given and what they had changed after the course.

**Results:**

Knowledge was essential for making lifestyle changes following education. Three factors affected whether lifestyle changes were implemented: obtaining new knowledge, taking responsibility, and receiving confirmation of an already healthy lifestyle. Four factors motivated individuals to maintain changes: support from others, experiencing an effect, fear of complications, and the formation of new habits.

**Conclusion:**

Knowledge was used to make and maintain changes in diet, medication and physical activity. Knowledge also acted as confirmation of an already adequate lifestyle. Knowledge led to no changes if diabetes appeared “not that scary” or if changes appeared too time consuming. Those involved in diabetes education need to be aware of the challenges in convincing asymptomatic patients about the benefits of adherence to self-management behaviour.

## Introduction

Type 2 diabetes mellitus (T2DM) is a chronic, progressive disease, and its impact on individuals, populations, and public health is increasing [1]. Having diabetes strongly influences daily life. Many patients worry about the chronicity of the disease and the possible complications, are insecure about living with their disease, and struggle with declining health and loss of function [2], [3]. This impacts patients' capacity to contribute to family and social life.

Diabetes self-management, such as healthy eating and physical activity, is crucial to achieve glycemic control, sustainable weight loss [4], and minimal use of insulin [5]. Patients with type 2 diabetes are therefore expected to take responsibility for managing their disease; an undertaking that is challenging and deeply embedded in the patients' daily lives [6]. Diabetes patient education is considered one of the cornerstones of effective diabetes care, and is intended to improve patients' health status and quality of life [7]. Diabetes Self-Management Education (DSME) intends to facilitate the knowledge, skills, and abilities necessary to achieve effective self-care behavior and necessary behavior changes [8].

Research has shown that DSME programs for patients with diabetes type 2 can be beneficial. A meta-analysis including studies investigating the effects of self-care management interventions (both in groups and individually) showed improvement of glycemic control [9]. In a systematic review of group education programs, both blood sugar measurements and knowledge was improved at 6 months, 12 months, and two years after the program [10]. Self-management skills and empowerment/self-efficacy was improved at 6 months. According to an Australian study diabetes education leads to a range of outcomes: knowledge and understanding, self-management, self-determination, psychological adjustment, clinical outcomes, and cost effectiveness [11]. The participants reported however a ranking where they considered knowledge and understanding as being most influenced from education, and cost effectiveness less influenced.

Although diabetes education is vital, research indicates that lifestyle changes and biomedical results might be difficult to maintain [9], [10]. Education is often not sufficient for patients to sustain a lifetime of diabetes self-care [12]. Many diabetes patients struggle to follow the advice they receive and their commitment to self-management decreases over time [13]. Support from diabetes specialist nurses, other patients, and family members is necessary to manage diabetes [14], [15]. Studies have also shown that goal setting becomes challenging when the supportive group environment disappears after the course [6], [15].

Further knowledge about the process of making and maintaining lifestyle changes is lacking. Knowledge about the period after participating in group-based DSME, and how changes are made and maintained, would be helpful in designing future educational courses and intervention research, as well as in work on public health. The aim of this study was therefore to investigate how participants make and maintain lifestyle changes after participating in group-based type 2 diabetes self-management education courses.

## Methods

### Ethics statement

The study was approved of by the regional committee for medical and health research ethics in Central Norway (4.2005.1556). All participants received oral and written information about confidentiality and anonymity, and signed a consent form before the interviews took place.

This was a qualitative interview study, conducted from November 2007 to May 2008 in a hospital trust in Central Norway providing specialized care at two hospitals to a population of 129.970 (2008). Qualitative interviews were chosen in order to explore in-depth the participants' experiences with making and maintaining lifestyle changes.

### Participants

Participants were recruited from seven diabetes self-management group education courses in two different hospitals in the same hospital trust. The education courses were locally developed and integrated into everyday practice. The groups consisted of 8–10 patients, usually referred from primary care. The courses lasted for 15 hours over three sessions, with one session per week. The courses were led by diabetes nurses, with input from physicians, physiotherapists and experienced patients. The courses included information about diabetes type 2, diet, physical activity, and metabolic control. The teaching methods employed were lectures with information and questions, interactive learning/skills training, and group discussions. The education courses are described in more detail in previous publications [16].

### Recruitment

The course leaders informed the participants about the interview study, and those willing to participate were contacted by the researcher. The sample of participants was thus a convenience sample; all participants in the education courses were invited, and those who volunteered took part in the interviews. The participants were adults with a confirmed type 2 diabetes diagnosis who had been to a GP consultation during the last three years. The participants had been diagnosed with diabetes type 2 between 1 month and 20 years before they participated in the education course. (More information on time since diagnosis is in table 1.) There was no pre-set cut-off level for A1C. Patients who had attended a diabetes education program during the last 12 months were excluded. The participants also took part in a qualitative study investigating the patients' expectations before the course [3], and some took part in a randomized controlled trial on the effect of group based diabetes self-management education [16].

**Table 1:** Description of participants and type of changes made (only participants in individual interviews, N = 15).

Age group	Gender	Time since diagnosis	Areas of change in the period after course *
30–39	Female	2 months	Diet, physical activity, confident.
40–49	Female	10 years	Diet, physical activity, new medication, confident, motivated.
	Female	6 years	Diet, physical activity, blood glucose testing, taken control.
	Male	1 month	Diet, physical activity, blood glucose testing, taken control.
50–59	Female	4 months	Diet, physical activity, blood glucose testing, responsibility, identity.
	Male	5 months	Diet, blood glucose testing, more interested.
	Male	10 years	Diet, physical activity, medication, responsibility.
	Male	7 months	No change, not interested.
	Male	1 year	Blood glucose testing, more serious.
60–69	Female	12 years	Diet, physical activity, taken control.
	Male	10 years	No change, not interested.
	Male	2 months	Diet, more serious.
	Male	4 years	Diet, physical activity, confident, motivated.
70–79	Female	20 years	Diet, physical activity, confident.
	Female	2 months	No change, confident, relaxed.

* Areas of change include physical activity, diet, monitoring blood glucose and attitude towards diagnosis.

### Data collection

Data was collected in two rounds through in-depth semi-structured focus groups and individual interviews, just after the course and six months afterwards. A mix of focus group and individual interviews was chosen since focus groups gave broadness and overview, while the individual interviews provided more depth. The 2^nd^ author conducted all interviews. A semi-structured interview guide was used [17]. The participants were asked how they had used the advices given in the course and what they had changed after participating. Initially, two focus-group interviews were conducted, consisting of 3 and 4 participants, respectively, from two education courses. This provided information about specific lifestyle changes and an overview of the relevant themes. This information was used to adjust the interview guide that was used in the individual interviews. The individual interviews investigated variations in the extent of participants' lifestyle changes, and provided more in-depth information about why and how the informants had made changes. Data collection ceased when saturation was achieved [18]. All interviews were audio recorded and transcribed verbatim. Demographics, time since diabetes diagnosis, and information about treatment were collected from all participants.

### Data analysis

Since the main topics in the focus group interviews and the individual interviews were similar, data from the two different types of interviews were combined and analyzed together. Data was analyzed by systematic text condensation [19], a qualitative method inspired by and based on psychological phenomenological analysis [20]. The method can be used to analyze interview data, field notes from observations, or written text, and is exploratory and descriptive. In accordance with the method of systematic text condensation, researchers began by reading the transcripts and annotating them with initial thoughts, in order to get a general sense of the whole and an idea of the main themes. The transcriptions were read once more and meaningful text elements were identified and systematized (coded) into the main themes. New themes evolving from the transcripts were added. In the third part of the analysis process, groups within themes were identified and sorted into subgroups. The text from the subgroups was then condensed into “artificial” citations (condensates of the content which are not real quotes, but aim at maintaining the terminology used by the participants). In the last part of the process an introduction to each subgroup was created. The themes were discussed among the authors to reduce bias. Some authors had experience as health professionals and some did not. Validity was checked by referring back to the original transcript and making sure the connection was evident. Coding of interviews was continued until topic saturation, when it was clear that no new themes or links emerged. The Norwegian citations were translated and edited into a more readable form by removing redundant words and pauses and changing the local accent into written language. The quotes presented in this article are typical of the views expressed by the participants and are used to exemplify and illustrate the themes. All quotes presented are from the individual interviews.

## Results and Discussion

Fourteen women and nine men participated in the interviews. Seven were interviewed in focus groups and 15 individually (table 1). Eighteen of the 23 participants were interviewed at both time points. The remaining five informants were interviewed only once due to practical obstacles, four right after the course and one six months afterwards. The mean age of the participants was 58 years (range 35–72). Fourteen out of 23 were using medications for type 2 diabetes.

### Making lifestyle changes

The participants reported a range of lifestyle changes after taking part in the self-management course (table 2). Knowledge was described as essential for making lifestyle changes after taking part in the course. Three factors affected whether or not lifestyle changes were implemented: obtaining new knowledge, taking responsibility for diabetes care, and receiving confirmation of an already healthy lifestyle.

**Table 2:** Lifestyle changes made by the informants (N = 23).

Areas	Type of change (no. of participant reports)
Self-monitoring of blood glucose levels	Measured after different types of food (6)
	Started to measure (5)
	Bought an electronic blood glucose meter (3)
	Measured more frequently (3)
	Measured after different physical exercise (1)
Physical activity	Increased physical activity (11)
	Used physical activity after big meals to normalize blood glucose level (2)
	Bicycled every day (2)
	Started at the gym (2)
	Used physical activity as a tool in controlling blood glucose (1)
	Walked the stairs (1)
	Started to swim (1)
Diet	Lower carbohydrates intake (13)
	Started reading food content declaration (8)
	Reduced fatty food (8)
	More wholegrain products (7)
	Stopped buying specific unhealthy products (5)
	Chosen food after how it influence on blood glucose (5)
	Increased vegetable and fruit intake (3)
	Smaller portions of unhealthy food (2)
	More regular meals (2)
	Less coffee and milk (1)
	Brought their own food to parties (1)
Other changes	Started on new medication (2)
	Enrolled in the local diabetes association (2)
	Quit smoking (1)
Attitude	Taken control (6)
	Safer/secure (5)
	More serious (4)
	More motivated (3)
	Taken responsibility (2)
	More relaxed (1)
	More interested in diabetes (1)
	Taken the “diabetic identity” (1).

#### Obtaining new knowledge

The participants said that they had obtained new knowledge was obtained in the course, which constituted one of the factors leading to lifestyle changes. This knowledge came from lectures by the diabetes educators, from a lecturer who had diabetes type 2 (an experienced patient), and from interactions with other participants.

Patients stated that eating healthily was easier after obtaining new knowledge about food and different ingredients.

*When I'm out grocery shopping I read the food labels. I have learned that. That makes it easier for me to choose food that is healthier in relation to the illness.*

(Male, 60–69 years, diagnosed four years ago).

New knowledge about how medication influences the disease also led to improved self-management behavior regarding medication. Some had started taking medications or changed the type of medication after the course.

*It* [the medicine] *was sort of a new consequence after the course. It made me more aware of the fact that pills are important to get the full benefit of the capacity of the pancreas. I was very motivated to start with that* [medication] *… this made it easier to understand.*

(Woman, 40–49 years, diagnosed 10 years ago).

The participants also expressed a desire to live a normal life. New knowledge had made them understand that they could live “a normal life” despite their condition. Information on how different foods affected glucose levels and how physical activity could regulate this was used to compensate for planned or unplanned dietary lapses.

*If I'm invited to a party in the evening, then I measure my blood sugar before I leave home. And if the blood sugar is high, just over 7, then I go jogging.*

(Male, 40–49 years, diagnosed one month ago).

However, new knowledge did not lead to lifestyle changes for all informants. A few informants said that making changes was not a priority. These informants were able to repeat accurately the basic recommendations and advice given at the course, but did not make changes. One of the informants found the information about food ingredients useful, but stated that it would not influence his choices when grocery shopping since he found it time consuming.

*I buy the same food products as before. But if I want to, I now have the opportunity to follow up better.*

(Male, 50–59 years, diagnosed 7 months ago).

New knowledge also led to the perception that diabetes was “not that scary” and made some consider self-management unnecessary. Knowledge about diet also led to insecurity for some of the participants and led to no lifestyle changes.

*I eat chocolate. I eat ice cream too. I also eat fat pork ribs. I eat lots of fruit, but that is also dangerous. It is sugar and yeast. So it's all the same what you eat. I choose what's on the shelf in front of the tip of my nose. Because whatever I'm looking for it contains something that shouldn't be there and there is no point in going from grocery store to grocery store searching for something suitable for my body.*

(Male, 50–59 years, diagnosed 7 months ago).

#### Taking responsibility

Some participants reported that lifestyle changes were a result of a changed understanding of their role and of taking responsibility for their own health.

*What has changed is that before the course I thought my doctor had the responsibility for all this. But now I understand that it's me. I have to make the decisions myself, and I feel that I have taken control. If the doctor was the one who had the responsibility, I would have eaten the wrong things because it wasn't my responsibility, right. But now the responsibility is mine and I have to deal with the consequences.*

(Woman, 50–59 years, diagnosed four months ago).

Nevertheless, a few of the participants said that someone else was responsible for managing and controlling the disease, like their doctor or their wife. These participants did not make lifestyle changes. One man said that he could not make changes in his diet because his wife was responsible for all the cooking.

*It's my wife who keeps track of what I eat. Cause she had diabetics in her family. Yes, I reckon that I eat what I'm permitted to eat.*

(Male, 60–69 years, diagnosed 10 years ago).

#### Getting confirmation of a healthy lifestyle

According to some of the participants, the course provided confirmation that their way of living was compatible with good diabetes self-management. Some participants responded that they felt confident in their current lifestyle, and that this was enough.

*I feel safe because I always tried to eat fairly healthy and correct and I've found out that I can eat what I've been eating through the years. And I've never been particularly fond of sweets.*

(Woman, 70–79 years, diagnosed two months ago).

### Maintaining lifestyle changes

According to the participants, four factors were central to maintaining lifestyle changes: getting support from others, experiencing the effect of making changes, fearing complications, and making the changes a habit.

#### Getting support from others

Support was important for maintaining lifestyle changes. Some of the informants who had permanently increased physical activity said that they had been supported by children, friends, partners and doctors.

*My daughter is dancing, so she's eager to be active during the summer holiday. She has taken me with her.*

(Woman, 40–49 years, diagnosed 10 years ago).

#### Experiencing the effect of lifestyle changes

Participants described motivation resulting from experiencing the effects of lifestyle changes such as weight loss, more energy, slowed progression of diabetes complications, and increased well-being. Some expressed that they felt the diagnosis had changed their life in a good way since they now felt healthier than they had before the diagnosis.

*Yes, I have lost weight. About 20 kg… I actually feel in much better shape now than before I became sick. I exercise and I have a healthy diet. [...] I work as much as before, but I have a different diet and more energy.*

(Male, 40–49 years, diagnosed one month ago).

#### Fearing complications

Some maintained their new lifestyle due to a fear of complications from the disease, consequences from an unhealthy lifestyle, or becoming dependent on medications.

*To try to delay it* [the complications] *as long as possible. That is my goal.*

(Woman, 50–59 years, diagnosed four months ago).

#### Making the changes a habit

The participants said that when physical activity, a healthy diet and blood glucose monitoring became habits they were no longer a choice. The lifestyle was already a part of their daily lives.

*The best is that it* [new diet and increased level of physical exercise] *is not something that I'm aware of anymore. It has become a habit… a healthy habit.*

(Male, 60–69 years, diagnosed four years ago).

### Strengths and limitations

The interviews took place within a restricted geographical area since the interviews were conducted in one hospital trust. This is a potential limitation of this study. However, the participants were recruited from seven different diabetes education groups and gender, age, type of medication/treatment and time since diagnosis varied. The participants' education, current treatment, or level of compliance with this treatment was not recorded or considered specifically. The number of interviews is broadly consistent with the prevailing trend among qualitative studies [17]. The follow-up period of six months was rather short. Lifestyle changes are more difficult to uphold over periods longer than six months, and longer follow-up studies should therefore be investigated. Only patients who participated in the whole course period were recruited to participate. Including patients who quit might have offered other considerations and perceptions. Most of the participants had made and maintained one or more behavioral change after the course. Including patients who had not successfully implemented behavior changes would give more information about the barriers to implementation.

### Discussion of results

The present study showed that knowledge was necessary for making lifestyle changes. Participants in similar educational courses have previously expressed benefit from and valued the opportunity to gain additional knowledge [21]. It has also been suggested previously that general knowledge about treatments and risk factors for chronic conditions correlates with compliance with lifestyle changes and medications [22], [23]. Farmer and colleagues advocate knowledge as an important prerequisite for compliance to medical therapy, stressing that patients' belief about their self-efficacy to perform specific actions to promote their health is affected by the knowledge they attain [24]. Some participants in the present study changed their attitudes towards medication after getting new knowledge about medications' influence on diabetes. Not surprisingly, previous studies have also shown that attitudes and beliefs about medications correlate with medication compliance [38]. Malpass and colleagues have also shown that new knowledge led patients with diabetes type 2 to use physical activity as a support for dietary changes and to control blood glucose levels [14]. This is congruent with the results of the present study. The present study thus supports the notion that gaining knowledge drives changes in attitudes and behavior.

On the other hand, many have argued that increased knowledge and insight do not automatically lead to behavior change [25]. Many theories of change have been put forth to explain how people change their behavior. According to one of these, the “stages of change”-model, there are several stages in which the individual has the necessary knowledge before any action is taken to make behavioral changes [25]. Similarly, Khunti and colleagues found that although a patient education program led to changes in health beliefs three years after taking part in the education, there were no effects on biomedical outcomes (such as blood sugar control) or lifestyle changes [26]. For some participants in the present study new knowledge was insufficient to induce lifestyle changes. Some of them had all the necessary knowledge, but refrained from deciding to make any changes. According to the “stages of change”-theory these participants are in the stage of pre-contemplation; they know what actions are necessary in order to make changes, but still have no intention to do so [25]. While knowledge and understanding is an important prerequisite for lifestyle changes, the pathways from education via knowledge to actual behavior change are more complex. Some argue that focusing only on the individual patient's way towards the desired behavior leaves out the context of what that behavior means and ignores structures of inequality in society [27].

For some of the participants, the new knowledge led to the perception that diabetes was “not that scary”. For these participants, the information apparently reduced the potential risks of the illness. Denial of illness or failure to recognize the risks and consequences of an asymptomatic condition is known to be a barrier against diabetes self-management [28]. In line with several other studies, the present study found that some patients with type 2 diabetes might not consider diabetes a serious disease with important consequences, even after having participated in an education program [21], [29], [30]. Some argue that health professionals should give clear messages to hinder patients from shirking responsibility for lifestyle changes [31]. Peel and colleagues found that receiving a type 2 diabetes diagnosis was perceived as less “shocking” than previously stated in literature [32]. With the prevalence of type 2 diabetes increasing and the diagnosis becoming more common, the potentially negative impact of poorly managed diabetes might be more difficult to communicate.

According to the participants in the present study, an important factor in lifestyle changes was the perception of responsibility for managing the disease. We are not the first to demonstrate that providing knowledge with a focus on the patient's responsibility is crucial for behavioral change. A qualitative study conducted in the UK suggested that group education sessions play an important role in leading participants to become “accepters”; i.e., more accepting of a changed identity and its implications for their disease management [21]. According to the “stages of change”-theory, taking action is a crucial step in the change process [25]. However, some of the participants in the present study did not take responsibility for making lifestyle changes. Others have also shown that patients take very different positions regarding responsibility; diabetes management ranged from taking full responsibility for the illness, self-management, and lifestyle changes to ascribing the illness to heritage and the management of the disease to medication, and leaving practical lifestyle changes to health professionals [31]. Education and knowledge does not necessarily lead to a perception of responsibility.

Although time since diagnosis was recorded, the present study did not focus specifically on the issue. However, the timing of information about illness and health behavior may be important. Peel and colleagues found that although previous literature has indicated that too much information at the time of diagnosis is unhelpful [33], patients wanted information as soon as possible after receiving a diagnosis [32]. Khunti and colleagues investigated long-term benefits three years after participants took part in an education program within only six weeks after diagnosis. They found no effects on health behavior, although psychosocial outcomes were improved 12 months after the program [26]. Thus, it is still unknown whether providing education programs closer to diagnosis would make lifestyle changes easier to make and maintain. This question should be investigated further.

The “stages of change”-model is one of the theories that try to explain how people change behavior [25]. Some argue that health professionals should help patients move through the stages of change, and that any intervention should be tailored to account for the stage of change that the patient has reached [34]. Several studies have shown that the patient's stage of change before undertaking treatment or lifestyle changes is closely linked to progress [25], [35]. In this view, the patient's current stage of change would be more important than time since diagnosis. This approach requires individual tailoring of patient education, which current group education programs have not been able to provide. Grouping patients according to these stages would allow for an individual approach that accounts for patients' readiness to make changes.

Participants in the present study used new knowledge to make lifestyle changes with regard to how they lived “a normal life”. A study of management strategies for individuals with chronic pain similarly shows that “finding a new normality“ was an important factor for finding new fulfillment in life despite the limitations of a health condition [36]. In the present study some participants used physical activity as a “tool” to compensate for dietary lapses so they could participate in social events that included food. Previous studies have similarly shown that increased physical activity can act as a gateway-behavior; i.e., behavior that produces positive effects on other behaviors [14], [37].

According to the participants, four factors acted as motivation to maintain the implemented lifestyle changes: getting support from others, experiencing the effect of making changes, fearing complications, and making the changes a habit. Malpass and colleagues' qualitative study also highlighted some of the same factors [14]. They found that support, experiencing the effects of a new lifestyle, and fear of complications motivated patients with type 2 diabetes to manage their disease.

The support that participants received was an important factor in their commitment to new lifestyle behaviors. Several studies have previously shown that continuous support from family and health professionals is essential for maintaining diabetes-related self-care behaviors [6], [38], [39]. Having a dog or a person to walk with can also be factors that support the maintenance of increased physical activity [40]. In general, establishing and using supportive relationships is described as an important process when making changes, and is linked to the stages of taking action and maintaining change [25].

Making lifestyle changes a habit was also an important factor in the maintenance of lifestyle changes in the present study. Peel and colleagues found that the establishment of a routine was important for patients with diabetes who succeeded in maintaining regular physical activity over time [40]. The “stages of change”-theory describes a spiral in which one constantly has to repeat the steps of preparation and action to maintain the change [25]. Since most people relapse [25], the establishment of habits is considered necessary to maintain the changes over time.

The participants who experienced the effect of lifestyle changes said that this made them more confident and more motivated to maintain the changed lifestyle. Gaining confidence in one's ability to be an active “self-manager” is important. Experiencing successes helps change individuals' perception of their capacity to self-manage [41], and the experience of successful self-management leads to a stronger feeling of capability and confidence [42]. Experiencing an effect and being more confident lead to even more success, through increased engagement in healthy behaviors. The experience of effective change also provides more positive emotions, making people more open to new information and to adopting new behavioral strategies [43]. This knowledge is congruent with the results from the present study.

Much of the research on lifestyle changes has focused on single behaviors, e. g., smoking cessation, and authors have argued that multiple behavioral changes, such as lifestyle changes for patients with type 2 diabetes, includes a greater number of influential factors than single behaviors [35]. Such multiple behaviors also require the person to make a number of simultaneous changes, i.e., quitting smoking while starting to exercise and balancing a healthy diet. It is reasonable to believe that lifestyle changes that require multiple behavioral changes are more difficult to accomplish. However, some research has shown that there are some factors that are common for different problem areas [35]. Type of treatment, current stage of change, severity of the problem (lower severity - more change), and degree of effort (more effort - more change) all predict the success of behavior changes.

## Conclusions

This study has shown that knowledge obtained during diabetes self-management education is used by the participants to make and maintain changes in diet, medication, and physical activity. Knowledge can also act as a confirmation that one's lifestyle is sufficiently healthy. However, knowledge led to no changes if diabetes appeared “not that scary” or if lifestyle changes appeared too time consuming. Those involved in diabetes education need to be aware of the challenges in convincing asymptomatic patients about the benefits of adherence to self-management behaviour. Long-term studies on the implementation and maintenance of lifestyle changes might highlight how diabetes self-management can be sustained. Further qualitative studies should explore the different stages of change in the process of altering health behaviours for those with type 2 diabetes.
